# Direct Measurement of Plant Cellulose Microfibril and Bundles in Native Cell Walls

**DOI:** 10.3389/fpls.2020.00479

**Published:** 2020-04-24

**Authors:** Bo Song, Shuai Zhao, Wei Shen, Cynthia Collings, Shi-You Ding

**Affiliations:** ^1^Department of Plant Biology, Michigan State University, East Lansing, MI, United States; ^2^Great Lakes Bioenergy Research Center, Michigan State University, East Lansing, MI, United States; ^3^State Key Laboratory for Conservation and Utilization of Subtropical Agro-bioresources, College of Life Science and Technology, Guangxi University, Nanning, China

**Keywords:** cellulose microfibril, atomic force microscopy, direct imaging, primary cell wall, secondary cell wall, cellulose synthesis

## Abstract

Plants use rigid cellulose together with non-cellulosic matrix polymers to build cell walls. Cellulose microfibrils comprise linear β(1,4)-glucan chains packed through inter- and intra-chain hydrogen-bonding networks and van der Waals forces. Due to its small size, the number of glucan chains and their arrangement in a microfibril remains elusive. Here we used atomic force microscopy (AFM) to directly image primary cell walls (PCWs) and secondary cell walls (SCWs) from fresh tissues of maize (*Zea mays*) under near-native conditions. By analyzing cellulose structure in different types of cell walls, we were able to measure the individual microfibrils in elongated PCWs at the sub-nanometer scale. The dimension of the microfibril was measured at 3.68 ± 0.13 nm in width and 2.25 ± 0.10 nm in height. By superimposing multiple AFM height profiles of these microfibrils, the overlay area representing the cross-section was estimated at 5.6 ± 0.4 nm^2^, which fitted well to an 18-chain model packed as six sheets with 234432 conformation. Interestingly we found in PCW, all these individual microfibrils could be traced back to a bundle in larger imaging area, suggesting cellulose are synthesized as large bundles in PCWs, and then split during cell expansion or elongation. In SCWs where cell growth has ceased we observed nearly-parallel twined or individual microfibrils that appeared to be embedded separately in the matrix polymers without the splitting effect, indicating different mechanisms of cellulose biosynthesis in PCW and SCW. The sub-nanometer structure of the microfibril presented here was measured exclusively from elongated PCWs, further study is required to verify if it represents the inherent structure synthesized by the cellulose synthase complex in PCWs and SCWs.

## Introduction

Plant growth and development relies on the regulation of cell wall biogenesis. As the main skeletal component, cellulose forms interwoven microfibril networks to constitute the multilayer (lamellae) architecture observed for plant cell walls (Somerville et al., 2004). During cell growth and development, the biosynthesis and dynamic arrangement of the cellulose microfibrils play a key role in maintaining the mechanical properties and physiological functions of the cell walls (Cosgrove, 2016; Zhang et al., 2017, 2019). Cellulose has relatively simple chemistry that comprises a number of linear homopolymeric chains of β(1,4)-D-glucosyl residues packed through intra- and inter-chain hydrogen bonding networks and van der Waals forces to form *para*-crystalline microfibrils. The native structures of cellulose have been determined based on non-plant sources of large (20–40 nm) cellulose crystals (Nishiyama et al., 2002, 2003). In plant however, the cellulose microfibril has a small cross-sectional dimension (2–3 nm), in which the number of chains and how they pack into a microfibril is unknown.

Traditional high-resolution imaging techniques, such as electron microscopy (McCann et al., 1990; Xu et al., 2006) and field emission SEM (Carpita et al., 2001a, b; Zheng et al., 2017), have been extensively used to measure the cellulose microfibrils in plants, resulting in diameters in a range of ~3–50 nm depending on cell wall types. This wide range of size distribution probably represents microfibril bundles that either exist in native cell walls or are formed during sample preparation (Carpita et al., 2001a, b; Ding and Himmel, 2006; Ding et al., 2012; Zhang et al., 2013, 2016, 2017; Zheng et al., 2017). Despite the daunting challenges of directly measuring the cellulose microfibril, analytic methods, such as nuclear magnetic resonance (NMR) and diffraction-based techniques, have been widely used to characterize the physicochemical properties of plant cellulose. Early works by Chanzy et al. (1978, 1979) proposed a 15–25-chain cellulose fibril based primarily on diffraction data. Newman et al. (2013) used various analytic approaches, such as solid-state NMR, small-angle X-ray scattering, synchrotron wide-angle X-ray scattering, and computer simulation techniques and proposed 18-chain models with mixed cross-sectional shapes and possible microfibril twinning in the cell wall. Controversially, a model containing at least 24-chains has also been proposed by other researchers based on analysis of similar techniques (Wang and Hong, 2016) when the microfibril twinning effect is not considered.

Native cellulose in plant cell walls often appear to be bundles with variable sizes and closely associated with hemicelluloses. Cellulose structure could also be continuously modified during cell expansion, elongation and cell wall thickening and lignification (Busse-Wicher et al., 2014). Analysis of cellulose often requires chemical treatment and/or dehydration processes, which further alter cellulose structure (O’Neill et al., 2017), it is therefore difficult to interpret the diffraction data and calculate the fundamental structure of a microfibril based on ensemble average measurement.

Discovery of cellulose synthase (*CESA*) genes in different plant species and biochemical studies of cellulose synthase complexes (CSC) (Kimura et al., 1999) have provided new insights into prediction of microfibril structure. Plant cellulose is synthesized in plasma membrane by multiprotein CSCs. Observations using freeze fracture electron microscopy (FF-TEM) (Mueller et al., 1976; Giddings et al., 1980; Mueller and Brown, 1980; Nixon et al., 2016) and immuno-EM (Kimura et al., 1999) have suggested that the CSC appears to be a six-lobed rosette containing multiple CESAs. It has been therefore postulated that the number of chains in a microfibril should be 6-fold, assuming the CSC comprises only active CESAs and each CESA synthesizes one cellulose chain, thus 18- (Jarvis, 2013; Newman et al., 2013; Nixon et al., 2016), 24- (Fernandes et al., 2011; Thomas et al., 2013; Wang and Hong, 2016) or 36-mer (Scheible et al., 2001; Doblin et al., 2002) models of CSCs and corresponding microfibril models containing 18-, 24-, and 36-chains, respectively have been proposed. Computational simulations (Oehme et al., 2015) and density functional theory calculations (Kubicki et al., 2018) have suggested that 18-chain is more favorable than 24- or 36-chain models (Haigler and Roberts, 2019).

Atomic force microscopy (AFM) uses a sharp tip to probe the surface features by raster scanning, which offers a non-destructive approach to characterize biological materials from cellular to molecular scales (Dufrene et al., 2017). The resolution of early AFM works was comparable to electron microscopy, and showed additional fine details of microfibril arrangement when imaging plant cell walls (Kirby et al., 1996). However the quality of an AFM image relies substantially upon the sharpness of the tip, firmness and flatness of the sample, and imaging environments. AFM technique has recently been greatly improved by the development of ultra-sharp probes (1–2 nm) combined with new operation modes for imaging in liquid (Pyne et al., 2014; Shiotari and Sugimoto, 2017; Wang et al., 2017), resulting in typically an order of magnitude enhancement of spatial resolution compared to that obtained in ambient conditions (Fukuma et al., 2007; Israelachvili, 2011; Voitchovsky, 2013; Miller et al., 2016). While imaging plant tissue, the quality of AFM images could be affected by the 3-dimensional structure of the cell wall at the cellular (micrometer) scale and the complex architecture at the molecular (nanometer) scale. Previous studies have shown that the sizes of the microfibril appear larger under dehydrated condition than that observed in water (Pesacreta et al., 1997; Thimm et al., 2000). Our early studies (Ding and Himmel, 2006; Ding et al., 2012, 2014) have revealed 3–5 nm microfibril, the uncertainty of measured dimension is likely due to over-estimation of the actual size under dehydrated condition. Recently, imaging cell walls in aqueous buffer have estimated the width of the cellulose microfibril at ~3.5 nm (Zhang et al., 2013, 2016, 2017). However, the quality of these published images has yet to be sufficient to resolve the dimension and the cross-sectional shape of the microfibril.

**FIGURE 1:**
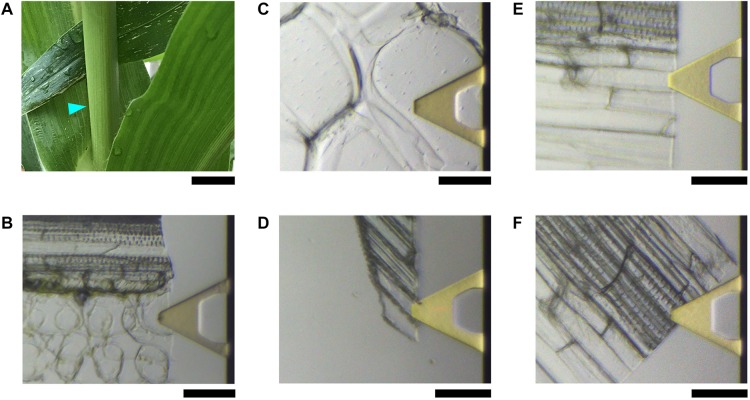
Maize plant and tissues used for AFM imaging. **(A)** Four-week vegetative growing maize plant. The cyan triangle indicates the approximate location of the third internode where the tissues containing a single layer of cells in **(B–F)** are obtained. Light microscopy of gold-plated (yellow) triangles in **(B–F)** are the AFM cantilever, in which the tip is located underneath; photos are taken in real-time during imaging in water showing the actual areas where AFM images are taken. **(B)** Mature Vascular bundle sheath cells that have thin primary cell wall (PCW). **(C)** Thickened and expanded PCW in stem pith parenchyma. **(D)** Thickened and elongated PCW in the rind fiber under the epidermis. **(E)** Secondary cell walls (SCWs) of fibers in vascular bundle. **(F)** SCWs in xylem vessel cells. Scale bar = 2 cm **(A)**, 50 μm **(B–F)**.

In this study, we took the advantages of AFM imaging in aqueous condition and a combined effort of sample preparation, pre-selection of tips and systematic adjustment of imaging parameters to optimize the quality of images. The primary cell walls (PCWs) and secondary cell walls (SCWs) from fresh tissues of maize (*Zea mays*) (Figure 1) were directly imaged and analyzed at the sub-nanometer scale.

## Results

### Optimization of AFM Image Quality

To minimize the potential alteration of cell wall structure, we used a double-edged razor blade to hand-cut fresh tissues longitudinally to yield ~5–10-μm slices containing a single layer of cells, and the sample was washed by water, mounted on a glass slide pre-coated by poly-lysine and then imaged in water (see section “Materials and Methods”). With the aid of in-line optical microscope, we positioned the AFM tip onto specific cell wall types (Figure 1) based on their morphological structures, so that different PCWs from expanded or elongated parenchyma cells, and SCWs from vessel and sclerenchyma fiber cells could be imaged reproducibly using different AFM tips and imaging parameters. We scanned initially in large sizes, i.e., 5 – 10 μm to localize areas of interest and “zoom-in” progressively from 1 μm to 100 nm with 512–1024 scan lines to allow observation of different scales from overall microfibril arrangement to sub-nanometer features of individual microfibrils. For each type of cell walls, we optimized the imaging process by altering scan sizes, scan rates, and applied forces, and repeatedly imaged the same area or the same type of walls but from independent sample preparations to compare the consistency of measured features. Using this strategy we could assess the tip quality with optimized parameter settings by means of identifying artifacts generated from the sample itself, environmental noise and mechanical drift.

**FIGURE 2:**
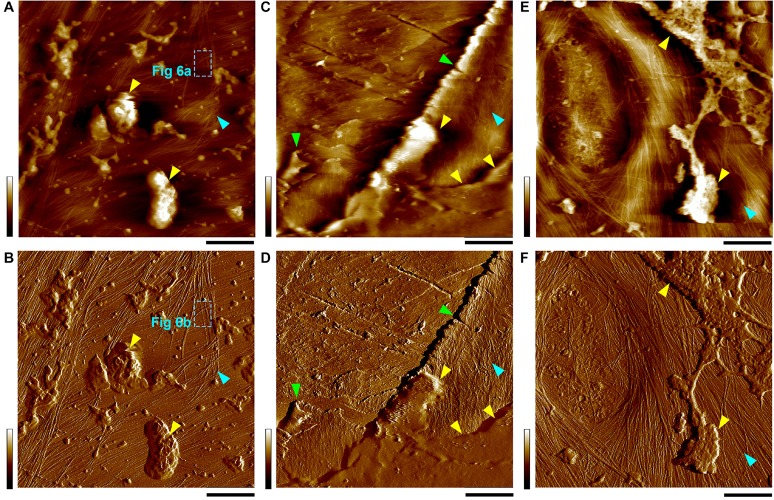
Height **(A,C,E)** and PeakForce error **(B,D,F)** images taken simultaneously in thickened PCWs. Yellow triangles indicate the amorphous substances, likely cytoplasmic debris that appears to be clearly distinguishable on wall surface, specifically in the PeakForce error images. Cyan triangles indicate the inner surface of the cell wall where cellulose microfibril networks can be observed. The boxes with cyan dashed lines **(A,B)** indicate the location of images in Figures 6A,B. Green triangles **(C,D)** Indicate the broken layers of the cell wall lamellae probably due to sample cutting during preparation. Occasionally, a primary pit field can also be observed **(E,F)**. Scale bar = 400 nm **(A,B,E,F)** and 2 μm **(C,D)**. Color bar = 100 nm **(A,E)**, 2 nN **(B)**, 200 nm **(C)**, 4 nN **(D)**, and 700 pN **(F)**.

In many cases, we observed particle shape features that appeared ultra-soft, presumably debris of cytosolic materials in large scan areas (Figure 2), which we intentionally excluded when imaging in small areas to reduce possible tip contamination and thus focused on tuning the imaging parameters optimized to observe fine details of the cellulose microfibrils.

**FIGURE 3:**
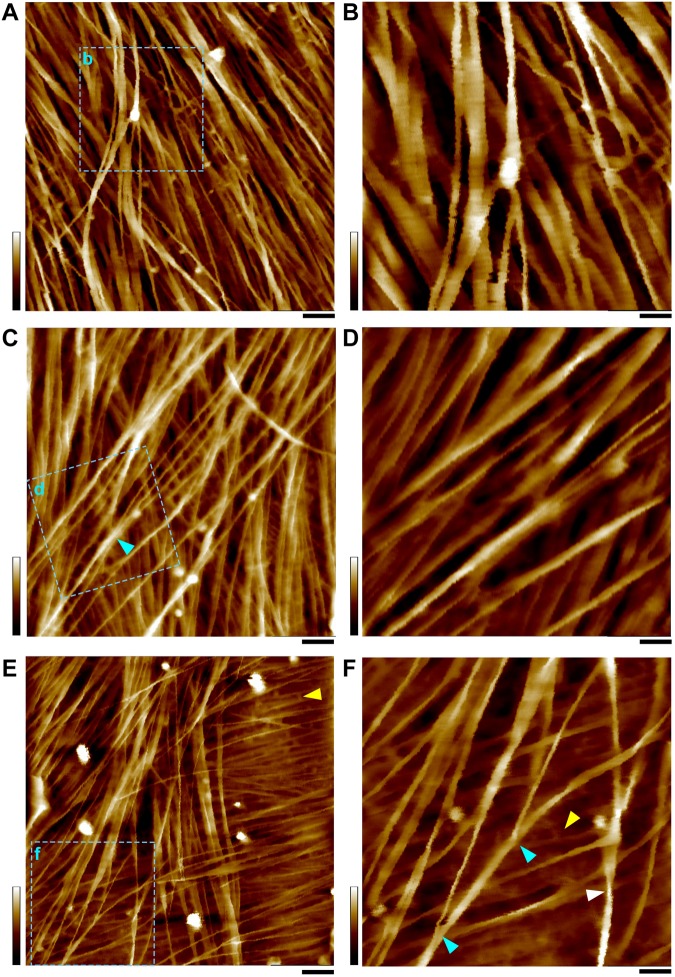
AFM height images of PCWs. **(A,B)** Thin and expanded PCW from bundle sheath cell (Figure 1B). **(C,D)** Thickened and expanded PCW from stem pith parenchyma (Figure 1C). **(E,F)** Thickened and elongated PCW from rind fiber (Figure 1D). The scan areas of **(B,D,F)** are indicated as boxes with cyan dashed-lines in **(A,C,E)**, respectively. Cell walls are prepared from the stem of living maize plant (Figure 1A) and imaged in water. A mixture of variable sizes of microfibril bundles are observed that may split into smaller bundles or individual microfibrils (cyan triangles in **C,F**). Amorphous substance (McCann et al., 1990) appeared to be matrix wall materials between microfibrils are indicated by yellow triangles **(E,F)**. Twisting microfibrils (white triangle in **F**) are found on wall surface. Scale bars = 50 nm **(A,C,E)** and 20 nm **(B,D,F)**. Color bars = 20 nm **(A,C,E)** and 15 nm **(B,D,F)**.

**FIGURE 4:**
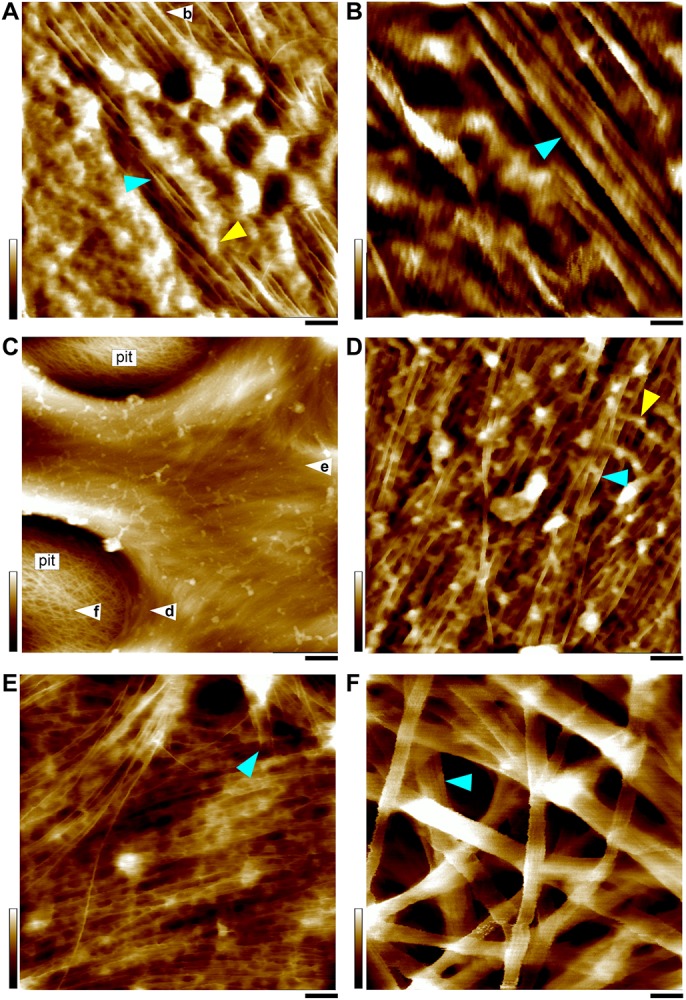
AFM height images of SCWs. **(A,B)** SCW of the fiber in the vascular bundle (Figure 1E). **(C–F)** Xylem vessel wall (Figure 1F). The SCWs contain predominately individual or twinned microfibrils indicated by cyan triangles in **(A,B,D,E)**, except the pit membrane area **(C,F)** that is the PCW containing large bundles. Apparent amorphous matrix components are indicated as yellow triangles in **(A,D)**. **(B)** “Zoom-in” image of the area in (white triangle in **A**), showing twinned microfibrils (cyan triangle). **(C)** The SCW between two large pits. **(D–F)** Are imaged from the areas indicated in (white triangles in **C**), respectively. **(D)** The microfibrils are imaged from the vertical wall of the pit cavity showing side view of twinned microfibrils. **(E)** The microfibrils in the surface are mostly parallel-arranged individual microfibrils and the termini are indicated by cyan triangle. **(F)** Large bundles (cyan triangle) in the pit membrane. Cell walls are prepared from the stem of a living maize plant (Figure 1A). Scale bars = 50 nm **(A,D,E,F)**, 20 nm **(B)**, and 500 nm **(C)**. Color bars = 15 nm **(A,D,E)**, 5 nm **(B)**, 20 nm **(F)**, 250 nm **(C)**.

The cell wall samples used in this study were only washed by water; it is assumed that cellulose microfibrils and matrix polymers, such as hemicelluloses and pectins are co-localized in native cell walls (Simmons et al., 2016). Previous studies have shown that the matrix polymers are not normally detectable by an AFM tip in liquid, due to the mobile nature of these polymers with the force applied by the probe (Zhang et al., 2016). Since the accuracy of AFM measurement is largely determined by the sufficient force required to gain imaging contrast without mechanical deformation of the sample (Leung et al., 2012), in this study we found the constant force less than 500 pN was critical to obtain high quality image showing sub-nanometer characteristics of the microfibril. At force ranging from 200 to 500 pN, we were able to obtain relatively sharp images of the microfibrils and minimized the effect of less-defined amorphous structures between the microfibrils (Figures 3, 4).

### Different Types of Cell Walls

Five types of cells were imaged extensively from fresh tissues of the stem of a vegetative growing maize plant (Figure 1A), including, (1) vascular bundle sheath cells (Figure 1B). Maize is a C4 plant, the bundle sheath cells contain chloroplast where the major step of photosynthesis, Calvin-Benson-Bassham cycle occurs to fix carbon into sugars. Therefore these expanded cells contain thin PCWs. (2) Pith parenchyma (Figure 1C). These cells normally expanded and have thickened PCWs. (3) Rind fiber cells immediately under the epidermis (Figure 1D). These cells are elongated and have thickened PCWs. (4) Sclerenchyma fibers adjacent to vascular bundle sheath (Figure 1E). These cells are elongated and have thickened lignified SCWs. (5) Vessels (Figure 1F). These cells are expanded and elongated and have thickened lignified SCWs.

In all observed PCWs cellulose appeared to be bundles with variable widths between 5 to 30 nm, which was consistent with previous studies (McCann et al., 1990; Carpita et al., 2001b; Ding and Himmel, 2006; Ding et al., 2012; Zhang et al., 2016). These large bundles split into small bundles and individual microfibrils (Figure 3). Some amorphous substances appeared to be bridging between microfibrils, which was similar with the observation by EM techniques (McCann et al., 1990). The SCWs were imaged from sclerenchyma fibers (Figures 1E, 4A,B) and xylem vessels (Figures 1F, 4C–F), in which predominately individual or twinned microfibrils were observed. The microfibrils in SCWs appeared to be near-parallel and independently embedded in the matrix polymers without further splitting effect (Figure 4). We found that such fibril splitting effect could be a signature feature to distinguish PCW and SCW, which is likely an indication of cell growth. We speculate that these large cellulose bundles are synthesized by multiple CSCs, which split as cell volume increases during cell expansion or elongation. All individual microfibrils observed in this study indeed could be traced back to a bundle of larger scales (Figure 3). The SCWs are deposited after cell growth has ceased, cellulose microfibrils are synthesized as individuals without further splitting.

The vessel wall is featured with large pits and pit cavities (Figure 4C). The pit membrane is formed before SCW deposition and is considered to be PCW, where only large bundles were observed (Figure 4F).

In the SCWs of the vessel, the microfibrils were observed both from the side (perpendicular) wall of the pit cavity (Figure 4D) and the surface (Figure 4E). The microfibrils appeared to be wider from the side view (Figure 4D) than those from the top view (Figure 4E), indicating that the cross-section of a microfibril is asymmetrical and the narrow side is vertically arranged on the wall surface (Figures 4B,D). Microfibril ends are also observed in the surface of SCWs (Figure 4E).

### Measurement of Individual Microfibrils

In survey of all AFM observations in 100–200 nm scan sizes from different types of cell walls, we found it was extremely difficult to measure the size and the cross-sectional shape of individual microfibrils. The measurement uncertainties include: (1) Bundling. In the PCWs, the overall structure of these bundles appeared to be ribbon-like and the size varied substantially in different walls. Although it was possible to estimate the numbers of microfibrils in a bundle based on its sequential splitting, the measured data was insufficient to calculate the accurate size of the microfibril due to unknown confirmation of these microfibrils in the bundle. (2) Dangling. During cell wall synthesis, cellulose microfibrils are deposited by layers and form complicated 3-dimentional networks. While the AFM tip detects the dangling microfibril with applied force, non-linear tip dilation artifacts may be generated thus increase the baseline noise. (3) Matrix polymers. The cell walls we imaged in this study were simply washed by water to minimize potential alteration of the native structure of the microfibril, however, the downside was the substantial amount of matrix polymers associated on the surface of the microfibril, especially in the SCWs (Figure 4), which could significantly increase the uncertainty of measuring the microfibril at the sub-nanometer scale. To address these issues, we developed several strategies to optimize measurement accuracy at the sub-nanometer scale.

We used pre-selected tips that were approximately 1 nm in radius and repeatedly imaged the same sample at least by three new tips to ensure image reproducibility in 100–200 nm scan scales. By examining the same microfibril imaged by different tips we found that the first couple of images appeared ultra-sharp when a new tip was used, and quickly became blurred in details after a few scans even though large features appeared to be the same, suggesting that in the case of imaging a small area it was possible that the geometry of the tip apex was critical rather than the overall tip size that could be easily worn out or contaminated (Santos et al., 2011). We further analyzed only the images taken by new tips.

**FIGURE 5:**
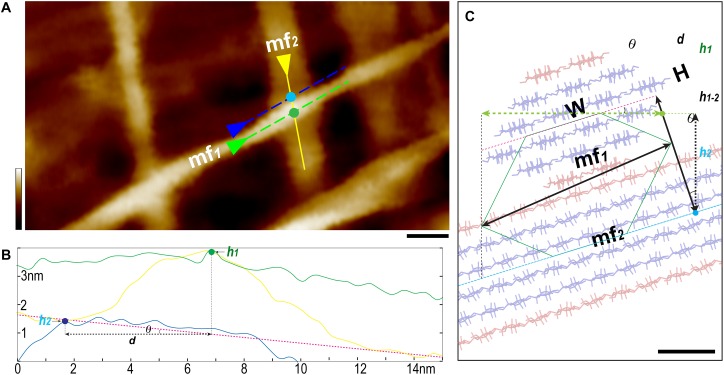
Three-line method to measure individual microfibril. **(A)** A typical AFM height image of thickened and elongated PCW scanned in 100 × 50 nm^2^ area with 1024 scan lines. Two well-defined microfibrils indicated as “mf*1* on the top of mf*2*” fashion are selected for measurement. **(B)** Using the section tool of AFM analysis software, three lines are drawn as shown in yellow, green and blue in **(A)** to generate height profiles. The *Rmax* value of mf*1* can be read out from the line profile (green line, **B**). **(C)** The 18-chain microfibril model (red-blue sticks, W, width; H, height) is used to show the relative arrangement of two microfibrils mf*1* (end view) and mf*2* (side view), red sticks present the hydrophobic surface chains. Based on this geometry, the height difference (*h*_1–2_) between mf*1* (measured height point *h*_1_, green dots in **A,B**) and mf*2* (measured height point *h*_2_, light blue dots in **A,B**) is calculated as *h*_1_ – *h*_2_, and the tilting angle *θ* of the mf*2* is determined by applying the first-order fitting (pink dash line in **B,C**). The distance (*d*) is between the two height-measuring lines (green and blue dash lines in **A**). The height of mf*1* is calculated as H = [h_1–2_+d*tan*(θ)*]* cos*(θ).* Scale bar = 10 nm **(A)** and 1 nm **(C)**. Color bar = 10 nm **(A)**.

By exploring the high quality AFM images, we found only in the case of a microfibril that run across the top of another microfibril (Figure 5A), thus provided a relative firm base locally to allow highly stable data acquisition, which could be found in the surface of elongated PCWs. We developed a simple three-line method (Figure 5) to measure the height and width of the microfibril based on raw image data.

**FIGURE 6:**
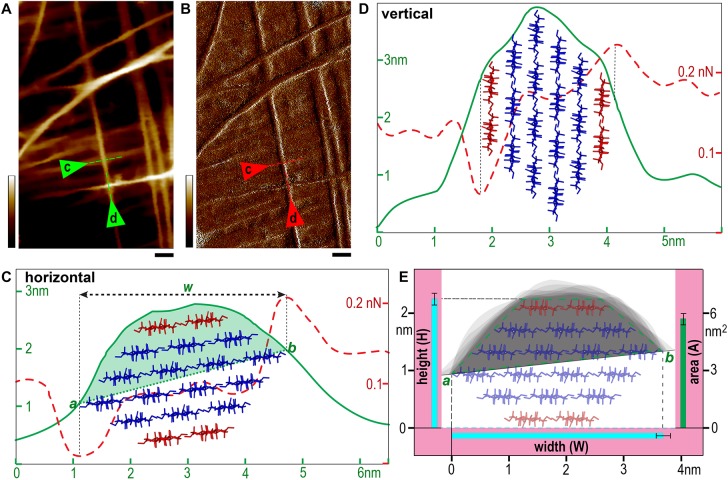
Microfibril measurement. **(A,B)** Typical height **(A)** and PeakForce error **(B)** images taken in 100 nm × 200 nm scan area. **(C,D)** Line profiles of two representative microfibrils indicated by triangles and dashed lines in **(A,B)** laid down on wall surface in nearly horizontal **(C)** or vertical **(D)** conformations. Profiles of heights are presented as solid green lines and PeakForce errors are dashed red lines. The edges of the microfibril (points *a* and *b* in the line profiles in **C**) are determined based on the spike PeakForce error signals (Andersson, 2006), and *w* indicates the horizontal distance. The half cross-sectional area of the microfibril (light green fill in **C**) is estimated between the line *a-b* and the height profile. **(E)** The heights (**H**), widths (W) and areas (**A**) are calculated using the three-line method described in Figure 5 presented as mean values with standard deviation (black bar) based on measured data from different microfibrils. The gray shaded area in (**E**, light green) indicates the overlay of multiple line profiles of individual microfibrils by aligning the points *a* and *b* in **(C)**. The data were measured based on a total of 40 raw images from different areas obtained from 30 sample preparations (only fresh sample and a new tip is used for each experiment). The proposed 18-chain microfibril model is showed as red (two chains in the hydrophobic surface) and blue sticks, which is used to show an empirical fit of the model to the experimental data **(C–E)**, and the half cross-sectional area is illustrated as a quadrilateral with green dashed lines in **(E)**. Scale bar = 20 nm **(A,B)**. Color bar = 10 nm **(A)** and 150 pN **(B)**.

The first line was drawn on the top of the target microfibril (Figure 5A, **mf*1***) along its long axis. The *Rmax* value (maximum vertical distance between the highest and lowest data points after the planefit) was read from the line profile of height image, which was used to determine if there were matrix polymers directly associated with the microfibril in the measured area. Considering the theoretical size of a sugar, such as a glucose molecule is 0.7–1 nm, the relatively mobile matrix polysaccharides could contribute at least 1 nm to the *Rmax*. If we choose the area that has *Rmax* smaller than 0.5 nm, it is plausible to assume the microfibril is clean cellulose. Our results suggest that in many cases the microfibrils are not fully covered by matrix polymers, especially these on the PCW surface (Figures 5, 6). Further measurement of the microfibril was only carried out in the area where apparently no matrix polymer was directly associated with the microfibril (*Rmax* < 0.5 nm).

The microfibril in the interwoven networks was often tilted with respect to the cell wall surface, which required a local plane correction. We drew a second line along the long axis of the bottom microfibril (Figure 5A, **mf*2***), and the third line that was parallel to but immediately adjacent to **mf*1***, so that the height (**H**) of **mf*1*** can be calculated based on the tilting angle (***θ***) and measured height values, respectively (Figures 5B,C).

It is known when imaging using tapping mode, such as PeakForce tapping used in this study, while the tip scans the edge of a feature (i.e., the microfibril), a momentary spike in the error signal appears before the controller can adjust the tip height. Therefore in a PeakForce error image that was taken simultaneously with the height image, the point of the spike could be used to estimate the edge of the feature (Andersson, 2006). Using this method, we drew a single line perpendicular across the microfibril in both height (Figure 6A) and PeakForce error (Figure 6B) images that were acquired simultaneously, and overlaid these two line profiles to determine the microfibril edges (Figure 6C, points ***a*** and ***b***), the measured width value (***w*)** could be read as horizontal distance between the point ***a*** and ***b***, and the actual width (**W**) could then be calculated based on the three-line method (Figure 5).

Previously we have found that the microfibril occasionally appeared twisted or laid down in different conformations on the wall (Figure 6), so that both height and width values of the same microfibril could be measured accurately based on height profiles (Ding et al., 2012). In this study, we further measured the microfibrils laid down on the wall surface in different conformations, i.e., horizontal (Figure 6C) vs. vertical (Figure 6D) in the same image, and the different line profiles suggest that the microfibril has an asymmetrical cross-sectional shape, which agreed with the observation in SCWs (Figures 4B,D).

Considering the AFM tip scanning on the surface of the cell walls only detects at maximum half of the microfibril surface – when the microfibril lies down in a nearly horizontal conformation (Figure 6C). We aligned and overlaid the edges (Figure 6C, points ***a*** and ***b***) of multiple height profiles, so that the cross-sectional area could be estimated (Figure 6E).

**FIGURE 7:**
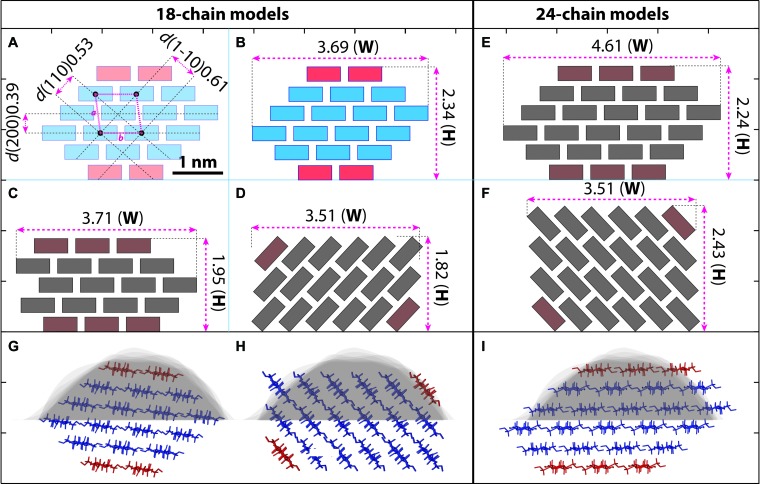
Hypothetical models of cellulose microfibril and empirical fitting to measured data. **(A–F)** Cross-section shapes and calculated height (H) and width (W) with 18-chain and 24-chain. Cellulose chains are presented as simplified boxes. **(A)** The 18-chain model is built based on the cellulose Iβ structure (Nishiyama et al., 2002). The two chains in the hydrophobic surface are shown in red. **(B–D)** Calculated size of the 18-chains 234432 model **(B)**, 34443 model **(C),** and 12333321 model **(D)**. **(E,F)** 24-chain models in 345543 **(E)** and 123444321 **(F)** conformations. **(G–I)** The 234432 model **(B)** fits the best to our AFM measurement (gray background, see also Figure 6E) compared with 12333321 **(D)** and 345543 **(E)** models. Scale bar = 1 nm.

Using these strategies, individual microfibrils were calculated with width (**W**), height (**H**) and cross-sectional area at 3.68 ± 0.13 nm (*n* = 33), 2.25 ± 0.10 nm (*n* = 63), and 5.6 ± 0.4 nm^2^ (*n* = 15), respectively. We then built microfibril models based on recent proposed models containing 18 or 24 chains (Fernandes et al., 2011; Jarvis, 2013; Newman et al., 2013; Thomas et al., 2013; Zhang et al., 2013; Nixon et al., 2016; Wang and Hong, 2016), assuming that plant microfibrils exhibit the same native cellulose Iβ structure (Nishiyama et al., 2002, 2003), and the chains have relatively regular arrangement. Theoretical heights and widths were estimated based on the conformations laid down on a surface (Figure 7). We found the 18-chain model arranged in 6-layer as 234432 (Figure 7G) fitted favorably into the data presented in this study (Figure 6E), compared with other 18-chain models, such as 34443 (Kubicki et al., 2018), 12333321 and 24-chain models.

## Discussion

Plant cellulose has been analyzed for decades by analytic methods and high resolution imaging approaches (Murdock, 1930; Ioyelovich, 1991; Wada et al., 2004; Agarwal et al., 2010; Barnette et al., 2011; Fernandes et al., 2011; Chunilall et al., 2013; Jarvis, 2013; Newman et al., 2013; Thomas et al., 2013; Wang and Hong, 2016). We and many other groups (Carpita et al., 2001a; Ding and Himmel, 2006; Ding et al., 2012; Zhang et al., 2013, 2016, 2017; Zheng et al., 2017) have demonstrated that the microfibrils often appear to be bundles especially in PCWs. Considering the facts that a mixture of bundles in variable sizes and individual microfibrils co-exists in different layers of any given cell wall, and the amount of matrix components may also affect the crystalline features of cellulose (Martinez-Sanz et al., 2017), the diffraction data measured from ensemble averaging of these mixed cellulose structures may not represent the fundamental structure of the microfibril. Indeed, previous studies (Fernandes et al., 2011; Jarvis, 2013; Thomas et al., 2013; Wang and Hong, 2016) have suggested 24-chain models by assuming that each microfibril is independent and has unique surface chains, however, they have also noted that an 18-chain model could fit into these data if the microfibrils coalesce even partly in their length without distinguishable surface chains.

We also noticed that even though the AFM operation conditions were extensively optimized in this study, most of images were still not suitable for determining the size and cross-section shape of the microfibril, because of the complicated three-dimensional architecture and the associated matrix components in native cell walls. We examined more than a thousand images taken from PCWs and SCWs, but we were only able to find a dozen of them that could be used to measure the microfibril at the sub-nanometer accuracy. Despite our data appeared to be preferable to the 18-chain 234432 model, which was measured exclusively from elongated PCWs, further study must be carried out to verify whether this microfibril structure is inherently synthesized by the CSC, or it is a resulting structure from splitting of large bundles.

In the SCWs, the microfibrils appeared to be twin or individuals with similar width, but the height value could not be accurately determined due to each microfibrils were independently embedded in the matrix materials (Figure 4). Although biochemical studies have shown that cellulose synthesis in the PCW and the SCW are carried out by different sets of CESAs (Taylor et al., 2003, 2004), the overall conserved sequences of these CESA proteins seem to suggest the same microfibril is produced in different walls. In this study the measurement of the microfibril has been performed in PCWs, the question remains unanswered if the same structure of microfibril is synthesized in SCWs as in PCWs. The large bundles and splitting effect of cellulose microfibrils observed in the PCWs are clearly different from the near-parallel twined or individuals in the SCWs, which suggest there are different mechanisms of CSC assembly during cellulose synthesis in different walls. Future studies on the structure and their molecular interactions between CESA proteins are required to better understand the fundamental mechanism of cellulose biosynthesis in plants.

## Materials and Methods

### General Chemicals

All chemicals and reagents, unless specifically noted, were purchased from Sigma–Aldrich (St. Louis, MO, United States).

### Preparation of Plant Cell Wall Material

For growth of maize plant, kernels of sweet corn seeds (Burpee Garden Products Company, #65681 Early Sunglow Hybrid) were directly sown in a pot filled with a mixture of humus soil, vermiculite, and perlite. The pot was placed in a growth chamber set to 30°C, 80% humidity, and a 12-h light/dark photoperiod. Maize plants were grown for 4 weeks prior to collecting tissues for further experiments.

For AFM imaging, we used the third internode of maize plants. All plant tissues were prepared by hand-cutting longitudinally using a double-blade razor. These sections were put in a petri dish with 20 ml ddH_2_O, washed at least three times by exchanging fresh water in the petri dish. These sections were then transferred onto a glass slide (EMS, Hatfield, PA, United States) or fresh-cut mica (Ted Pella, Redding, CA, United States) that was pre-coated with poly-lysine and checked by bright field light microscopy to select samples with a relatively uniform thickness of approximately 5–10 μm containing a single layer of cells (Figure 1). Extra water was then carefully removed by filter paper, and 100 μl fresh water was added immediately. The sample was allowed to settle down in water for at least 30 min in room temperature before AFM imaging. To ensure reproducibility, all samples were prepared from fresh plant tissue and discarded after imaging. In order to image all different types of cell walls from living plant, we grew at least 10 plants every week continuously in last the 3 years, at least a hundred plants have been used to generate the AFM images reported in this paper.

### AFM Operation

We used two AFM systems: The Dimension FastScan and the MultiMode 8-HR with NanoScope V controller (Bruker Nano, Santa Barbara, CA, United States). Both microscopes were installed with a vibration and acoustic isolation system. These two systems were used to compare imaging of the same cell wall sample as a quality control for fine features; we found when imaging in the scale of 100–200 nm scan scales using the same controller and imaging mode, the image quality was not distinguishable. Since the FastScan system has an open stage that allows for navigating the scanner in a large range, the entire cell wall surface can be imaged sequentially. Therefore, the majority of the image data presented in this study was carried out using the FastScan system.

The Fastscan AFM system allowed us to scan a field of up to 25 μm and the entire cell wall could be imaged by moving the sample stage (10–100 μm depending on wall type).

A standard 35-μm scanner was used with the ScanAsyst^TM^ imaging mode and probes SCANASYST-FLUID+ (Bruker, Camarillo, CA, United States) for imaging under fluid. The AFM control software (Nanoscope V9.3) was used in all imaging experiments. The PeakForce was manually controlled in values between 50 pN to 3 nN depending on surface features and the gain was automatically adjusted. Before AFM imaging, the scanner was carefully calibrated using calibration standards (Bruker, Camarillo, CA, United States) for x/y and z direction respectively. The system was warmed up for at least 2 h before imaging to minimize the creep phenomenon of the AFM scanner. During imaging, the x-y closed loop was always on to avoid image distortion caused by the hysteresis effect. A built-in optics system with a digital camera (5MP) was used to aid the positioning of the AFM tip to a desirable location and types of cell walls (Figure 1). Plant cell wall samples were kept in water during AFM imaging, images were taken at 512 × 512 and 1024 × 1024 lines with a scan rate of 0.5–3 Hz. At least five images with different scan sizes of 0.1, 0.2, 0.5, 1, and 5 μm, sometimes 10–20 μm if needed, were taken in the same scan area and on the same piece of cell wall, and at least five different areas were measured, both height and PeakForce error images were recorded simultaneously.

The cantilever of AFM probe (SCANASYST-FLUID+) was 70 μm long, 10 μm wide, and 600 nm thick. The spring constant is 0.7 N/m, and their resonant frequency in an aqueous solution is 150 KHz. The average tip radius was 2 nm. We preselected the AFM tips using the built-in function of “Tip Qualification” from the software Nanoscope Analysis v1.8 (Bruker Nano, Santa Barbara, CA, United States) to check the tip radius, and only the probes with tip radius of less than 2 nm were selected for the imaging experiment.

AFM provides a 3-D profile by raster scanning and recording the small interaction forces between a sharp tip and the sample surface. An AFM image therefore represents combined information of the tip geometry and the actual surface features of the sample (Santos et al., 2011). In this study, image deconvolution is particularly difficult due to the 3-D complexity of the relatively stiff cellulose networks and the surrounding matrix polymers that are highly mobile. In addition, the intrinsic resolution limit of AFM in imaging small features (smaller than the tip radius) can cause an overestimation of the width due to the tip broadening effect, and an underestimation of the height (height loss) due to sample deformation or intrinsic signal spread-out by the interaction of tip-surface-sample geometry (Santos et al., 2011).

A recently developed rapid force-distance (FD) curve-based imaging mode, PeakForce Tapping^TM^, is applied for minimizing the height loss, which allows precise control of probe-to-sample interaction and provides the lowest available imaging forces to achieve the highest resolution imaging (Pyne et al., 2014). Compared with regular AFM imaging technique, such as non-contact mode, the PeakForce tapping mode minimizes the height loss effect caused by tip-sample-surface interaction (Santos et al., 2011) by maintaining a constant contact force at the pN level which is suitable for the measurement of biological samples owing to its exceptional low imaging forces.

We adjusted the setpoint of contact force to be the smallest value as long as the images were reproducible to minimize the sample compression or deformation during imaging. The contact force may affect the measurement accuracy in both vertical and lateral directions, but minimal contact force with sharp image is likely to get the AFM measurement close to its actual value (Pyne et al., 2014). In our experiment, we found that by applying the PeakForce setpoint between 50 to 230 pN, most of cell walls could be imaged in high resolution, except scanning large areas, such as 5–10 μm, in which the force could be increased to a range of 200-3 nN.

### Measurement of the Height and Width of the Microfibrils

The software Nanoscope Analysis v1.8 (Bruker Nano, Santa Barbara, CA, United States) was used for AFM image processing and analysis. The height and PeakForce error images were analyzed, which were flattened at 3rd order and filtered with the lowpass filter (filter size less than 3 pixels) for images presented in all figures. The data scale was also manually adjusted according to the color bars presented in each image. For height and width measurement, we used only raw images in 100–200 nm scan areas with 1024 scan lines, no off-line flatten or filter were applied.

## Data Availability Statement

All data are available from the corresponding author upon reasonable request.

## Author Contributions

S-YD conceptualized the project, conducted AFM, analyzed the data, and wrote the manuscript. BS, SZ, WS, and CC conducted AFM and data analysis. All authors revised the manuscript.

## Conflict of Interest

The authors declare that the research was conducted in the absence of any commercial or financial relationships that could be construed as a potential conflict of interest.

## References

[B1] Agarwal U. P. Reiner R. S. Ralph S. A. (2010). Cellulose I crystallinity determination using FT–Raman spectroscopy: univariate and multivariate methods. *Cellulose* 17 721–733. 10.1021/jf304465k 23241140

[B2] Andersson S. B. (2006). “An algorithm for boundary tracking in AFM,” in *Proceedings of the American Control Conference 2006*, (Minneapolis, MN: IEEE), 508–513.

[B3] Barnette A. L. Bradley L. C. Veres B. D. Schreiner E. P. Park Y. B. Park J. (2011). Selective detection of crystalline cellulose in plant cell walls with sum-frequency-generation (SFG) vibration spectroscopy. *Biomacromolecules* 12 2434–2439. 10.1021/bm200518n 21615075

[B4] Busse-Wicher M. Gomes T. C. F. Tryfona T. Nikolovski N. Stott K. Grantham N. J. (2014). The pattern of xylan acetylation suggests xylan may interact with cellulose microfibrils as a twofold helical screw in the secondary plant cell wall of *Arabidopsis thaliana*. *Plant Journal* 79 492–506. 10.1111/tpj.12575 24889696 PMC4140553

[B5] Carpita N. C. Defernez M. Findlay K. Wells B. Shoue D. A. Catchpole G. (2001a). Cell wall architecture of the elongating maize coleoptile. *Plant Physiol.* 127 551–565. 10.1104/pp.010146 11598229 PMC125090

[B6] Carpita N. C. Defernez M. Findlay K. Wells B. Shoue D. A. Catchpole G. (2001b). Cell wall architecture of the elongating maize coleoptile. *Plant Physiol.* 127 551–565. 11598229 PMC125090

[B7] Chanzy H. Imada K. Mollard A. Vuong R. Barnoud F. (1979). Crystallographic aspects of sub-elementary cellulose fibrils occurring in the wall of rose cells cultured in vitro. *Protoplasma* 100 317–322.

[B8] Chanzy H. Imada K. Vuong R. (1978). Electron diffraction from the primary wall of cotton fibers. *Protoplasma* 94 299–306. 10.1007/bf01276778

[B9] Chunilall V. Bush T. Larsson P. T. (2013). “Supra-molecular structure and chemical reactivity of cellulose I studied using CP / MAS 13 C-NMR,” in *Cellulose - Fundamental Aspects*, (New York, NY: Intech).

[B10] Cosgrove D. J. (2016). Plant cell wall extensibility: connecting plant cell growth with cell wall structure, mechanics, and the action of wall-modifying enzymes. *J. Exp. Bot.* 67 463–476. 10.1093/jxb/erv511 26608646

[B11] Ding S. Y. Himmel M. E. (2006). The maize primary cell wall microfibril: A new model derived from direct visualization. *J. Agric. Food Chem.* 54 597–606. 10.1021/jf051851z 16448156

[B12] Ding S. Y. Liu Y. S. Zeng Y. N. Himmel M. E. Baker J. O. Bayer E. A. (2012). How does plant cell wall nanoscale architecture correlate with enzymatic digestibility? *Science* 338 1055–1060. 10.1126/science.1227491 23180856

[B13] Ding S. Y. Zhao S. Zeng Y. N. (2014). Size, shape, and arrangement of native cellulose fibrils in maize cell walls. *Cellulose* 21 863–871. 10.1007/s10570-013-0147-5

[B14] Doblin M. S. Kurek I. Jacob-Wilk D. Delmer D. P. (2002). Cellulose biosynthesis in plants: from genes to rosettes. *Plant Cell Physiol.* 43 1407–1420. 10.1093/pcp/pcf164 12514238

[B15] Dufrene Y. F. Ando T. Garcia R. Alsteens D. Martinez-Martin D. Engel A. (2017). Imaging modes of atomic force microscopy for application in molecular and cell biology. *Nat. Nanotechnol.* 12 295–307. 10.1038/nnano.2017.45 28383040

[B16] Fernandes A. N. Thomas L. H. Altaner C. M. Callow P. Forsyth V. T. Apperley D. C. (2011). Nanostructure of cellulose microfibrils in spruce wood. *Proc. Natl. Acad. Sci. U.S.A.* 108 E1195–E1203. 10.1073/pnas.1108942108 22065760 PMC3223458

[B17] Fukuma T. Higgins M. J. Jarvis S. P. (2007). Direct imaging of individual intrinsic hydration layers on lipid bilayers at Angstrom resolution. *Biophys. J.* 92 3603–3609. 10.1529/biophysj.106.100651 17325013 PMC1853155

[B18] Giddings T. H. Jr. Brower D. L. Staehelin L. A. (1980). Visualization of particle complexes in the plasma membrane of Micrasterias denticulata associated with the formation of cellulose fibrils in primary and secondary cell walls. *J. Cell Biol.* 84 327–339. 10.1083/jcb.84.2.327 7189756 PMC2110545

[B19] Haigler C. H. Roberts A. W. (2019). Structure/function relationships in the rosette cellulose synthesis complex illuminated by an evolutionary perspective. *Cellulose* 26 227–247. 10.1007/s10570-018-2157-9

[B20] Ioyelovich M. Y. (1991). Supermolecular structure of native and isolated cellulose. *Vysokomolekulyarnye Soedineniya Seriya A* 33 1786–1792.

[B21] Israelachvili J. N. (2011). *Intermolecular and Surface Forces*, 3rd Edn Cambridge, MA: Academic Press, 1–674.

[B22] Jarvis M. C. (2013). Cellulose biosynthesis: counting the chains. *Plant Physiol.* 163 1485–1486. 10.1104/pp.113.231092 24296786 PMC3850196

[B23] Kimura S. Laosinchai W. Itoh T. Cui X. Linder C. R. Brown R. M. (1999). Immunogold labeling of rosette terminal cellulose-synthesizing complexes in the vascular plant vigna angularis. *Plant Cell* 11 2075–2086. 10559435 10.1105/tpc.11.11.2075 PMC144118

[B24] Kirby A. R. Gunning A. P. Waldron K. W. Morris V. J. Ng A. (1996). Visualization of plant cell walls by atomic force microscopy. *Biophys. J.* 70 1138–1143. 10.1016/s0006-3495(96)79708-4 8785273 PMC1225043

[B25] Kubicki J. D. Yang H. Sawada D. O’Neill H. Oehme D. Cosgrove D. (2018). The shape of native plant cellulose microfibrils. *Sci Rep* 8 13983. 10.1038/s41598-018-32211-w 30228280 PMC6143632

[B26] Leung C. Bestembayeva A. Thorogate R. Stinson J. Pyne A. Marcovich C. (2012). Atomic force microscopy with nanoscale cantilevers resolves different structural conformations of the DNA double helix. *Nano Letters* 12 3846–3850. 10.1021/nl301857p 22731615

[B27] Martinez-Sanz M. Pettolino F. Flanagan B. Gidley M. J. Gilbert E. P. (2017). Structure of cellulose microfibrils in mature cotton fibres. *Carbohydr Polym* 175 450–463. 10.1016/j.carbpol.2017.07.090 28917888

[B28] McCann M. C. Wells B. Roberts K. (1990). Direct visualization of cross-links in the primary plant-cell wall. *J. Cell Sci.* 96 323–334.

[B29] Miller E. J. Trewby W. Payam A. F. Piantanida L. Cafolla C. Voitchovsky K. (2016). Sub-nanometer resolution imaging with amplitude-modulation atomic force microscopy in liquid. *J. Vis. Exp.* 118 54924. 10.3791/54924 PMC5226432 28060262

[B30] Mueller S. C. Brown R. M. (1980). Evidence for an intramembrane component associated with a cellulose microfibril-synthesizing complex in higher plants. *J. Cell Biol.* 84 315–326. 10.1083/jcb.84.2.315 7189755 PMC2110546

[B31] Mueller S. C. Brown R. M. Scott T. K. (1976). Cellulosic microfibrils - nascent stages of synthesis in a higher plant-cell. *Science* 194 949–951. 10.1126/science.194.4268.949 17748556

[B32] Murdock C. C. (1930). The form of the x-ray diffraction bands for regular crystals of colloidal size. *Physical Review* 35 8–23. 10.1103/physrev.35.8

[B33] Newman R. H. Hill S. J. Harris P. J. (2013). Wide-angle x-ray scattering and solid-state nuclear magnetic resonance data combined to test models for cellulose microfibrils in mung bean cell walls. *Plant Physiol.* 163 1558–1567. 10.1104/pp.113.228262 24154621 PMC3846134

[B34] Nishiyama Y. Langan P. Chanzy H. (2002). Crystal structure and hydrogen-bonding system in cellulose 1 beta from synchrotron X-ray and neutron fiber diffraction. *J. Am. Chem. Soc.* 124 9074–9082. 10.1021/ja0257319 12149011

[B35] Nishiyama Y. Sugiyama J. Chanzy H. Langan P. (2003). Crystal structure and hydrogen bonding system in cellulose I(alpha) from synchrotron X-ray and neutron fiber diffraction. *J. Am. Chem. Soc.* 125 14300–14306. 10.1021/ja037055w 14624578

[B36] Nixon B. T. Mansouri K. Singh A. Du J. Davis J. K. Lee J. G. (2016). Comparative structural and computational analysis supports eighteen cellulose synthases in the plant cellulose synthesis complex. *Sci Rep* 6 28696. 10.1038/srep28696 27345599 PMC4921827

[B37] Oehme D. P. Downton M. T. Doblin M. S. Wagner J. Gidley M. J. Bacic A. (2015). Unique aspects of the structure and dynamics of elementary Ibeta cellulose microfibrils revealed by computational simulations. *Plant Physiol.* 168 3–17. 10.1104/pp.114.254664 25786828 PMC4424011

[B38] O’Neill H. Pingali S. V. Petridis L. He J. Mamontov E. Hong L. (2017). Dynamics of water bound to crystalline cellulose. *Sci Rep* 7 11840. 10.1038/s41598-017-12035-w 28928470 PMC5605533

[B39] Pesacreta T. C. Carlson L. C. Triplett B. A. (1997). Atomic force microscopy of cotton fiber cell wall surfaces in air and water: Quantitative and qualitative aspects. *Planta* 202 435–442. 10.1007/s004250050147

[B40] Pyne A. Thompson R. Leung C. Roy D. Hoogenboom B. W. (2014). Single-Molecule Reconstruction of Oligonucleotide Secondary Structure by Atomic Force Microscopy. *Small* 10 3257–3261. 10.1002/smll.201400265 24740866

[B41] Santos S. Barcons V. Christenson H. K. Font J. Thomson N. H. (2011). The intrinsic resolution limit in the atomic force microscope: implications for heights of nano-scale features. *PLoS ONE* 6 e23821. 10.1371/journal.pone.0023821 21912608 PMC3166059

[B42] Scheible W. R. Eshed R. Richmond T. Delmer D. Somerville C. (2001). Modifications of cellulose synthase confer resistance to isoxaben and thiazolidinone herbicides in Arabidopsis Ixr1 mutants. *Proc. Natl. Acad. Sci. USA* 98 10079–10084. 10.1073/pnas.191361598 11517344 PMC56918

[B43] Shiotari A. Sugimoto Y. (2017). Ultrahigh-resolution imaging of water networks by atomic force microscopy. *Nat. Commun.* 8 14313. 10.1038/ncomms14313 28155856 PMC5296746

[B44] Simmons T. J. Mortimer J. C. Bernardinelli O. D. Poppler A. C. Brown S. P. Deazevedo E. R. (2016). Folding of xylan onto cellulose fibrils in plant cell walls revealed by solid-state NMR. *Nat. Commun.* 7 13902. 10.1038/ncomms13902 PMC5187587 28000667

[B45] Somerville C. Bauer S. Brininstool G. Facette M. Hamann T. Milne J. (2004). Toward a systems approach to understanding plant-cell walls. *Science* 306 2206–2211. 10.1126/science.1102765 15618507

[B46] Taylor N. G. Gardiner J. C. Whiteman R. Turner S. R. (2004). Cellulose synthesis in the Arabidopsis secondary cell wall. *Cellulose* 11 329–338. 10.1023/b:cell.0000046405.11326.a8

[B47] Taylor N. G. Howells R. M. Huttly A. K. Vickers K. Turner S. R. (2003). Interactions among three distinct CesA proteins essential for cellulose synthesis. *Proc. Natl. Acad. Sci. U.S.A.* 100 1450–1455. 10.1073/pnas.0337628100 12538856 PMC298793

[B48] Thimm J. C. Burritt D. J. Ducker W. A. Melton L. D. (2000). Celery (*Apium graveolens* L.) parenchyma cell walls examined by atomic force microscopy: effect of dehydration on cellulose microfibrils. *Planta* 212 25–32. 10.1007/s004250000359 11219580

[B49] Thomas L. H. Forsyth V. T. Sturcova A. Kennedy C. J. May R. P. Altaner C. M. (2013). Structure of cellulose microfibrils in primary cell walls from collenchyma. *Plant Physiol.* 161 465–476. 10.1104/pp.112.206359 23175754 PMC3532275

[B50] Voitchovsky K. (2013). Anharmonicity, solvation forces, and resolution in atomic force microscopy at the solid-liquid interface. *Phys Rev E Stat Nonlin Soft Matter Phys* 88 022407. 10.1103/PhysRevE.88.022407 24032849

[B51] Wada M. Chanzy H. Nishiyama Y. Langan P. (2004). Cellulose III I crystal structure and hydrogen bonding by synchrotron X-ray and neutron fiber diffraction. *Macromolecules* 37 8548–8555. 10.1021/ma0485585

[B52] Wang L. Wang H. Wagner M. Yan Y. Jakob D. S. Xu X. G. (2017). Nanoscale simultaneous chemical and mechanical imaging via peak force infrared microscopy. *Sci Adv* 3 e1700255. 10.1126/sciadv.1700255 28691096 PMC5482550

[B53] Wang T. Hong M. (2016). Solid-state NMR investigations of cellulose structure and interactions with matrix polysaccharides in plant primary cell walls. *J. Exp. Bot.* 67 503–514. 10.1093/jxb/erv416 26355148 PMC6280985

[B54] Xu P. Donaldson L. A. Gergely Z. R. Staehelin L. A. (2006). Dual-axis electron tomography: a new approach for investigating the spatial organization of wood cellulose microfibrils. *Wood Science and Technology* 41 101–116. 10.1007/s00226-006-0088-3

[B55] Zhang T. Mahgsoudy-Louyeh S. Tittmann B. Cosgrove D. J. (2013). Visualization of the nanoscale pattern of recently-deposited cellulose microfibrils and matrix materials in never-dried primary walls of the onion epidermis. *Cellulose* 21 853–862. 10.1007/s10570-013-9996-1

[B56] Zhang T. Tang H. Vavylonis D. Cosgrove D. J. (2019). Disentangling loosening from softening: insights into primary cell wall structure. *Plant J.* 100 1101–1117. 10.1111/tpj.14519 31469935

[B57] Zhang T. Vavylonis D. Durachko D. M. Cosgrove D. J. (2017). Nanoscale movements of cellulose microfibrils in primary cell walls. *Nat Plants* 3 17056. 10.1038/nplants.2017.56 28452988 PMC5478883

[B58] Zhang T. Zheng Y. Cosgrove D. J. (2016). Spatial organization of cellulose microfibrils and matrix polysaccharides in primary plant cell walls as imaged by multichannel atomic force microscopy. *Plant J.* 85 179–192. 10.1111/tpj.13102 26676644

[B59] Zheng Y. Cosgrove D. J. Ning G. (2017). High-Resolution Field Emission Scanning Electron Microscopy (FESEM) Imaging of Cellulose Microfibril Organization in Plant Primary Cell Walls. *Microsc Microanal* 23 1048–1054. 10.1017/s143192761701251x 28835298

